# III–V nanowires on black silicon and low-temperature growth of self-catalyzed rectangular InAs NWs

**DOI:** 10.1038/s41598-018-24665-9

**Published:** 2018-04-23

**Authors:** Tuomas Haggren, Vladislav Khayrudinov, Veer Dhaka, Hua Jiang, Ali Shah, Maria Kim, Harri Lipsanen

**Affiliations:** 1 Department of Electronics and Nanoengineering, Aalto University, Espoo, P.O. Box 13500, FI-00076 Finland; 2 Department of Applied Physics and Nanomicroscopy Center, Aalto University, Espoo, P.O. Box 15100, FI-00076 Finland

## Abstract

We report the use of black silicon (bSi) as a growth platform for III–V nanowires (NWs), which enables low reflectance over a broad wavelength range as well as fabrication of optoelectronic devices by metalorganic vapor phase epitaxy. In addition, a new isolated growth regime is reported for self-catalyzed InAs NWs at record-low temperatures of 280 °C–365 °C, where consistently rectangular [-211]-oriented NWs are obtained. The bSi substrate is shown to support the growth of additionally GaAs and InP NWs, as well as heterostructured NWs. As seed particles, both *ex-situ* deposited Au nanoparticles and *in-situ* deposited In droplets are shown feasible. Particularly the InAs NWs with low band gap energy are used to extend low-reflectivity wavelength region into infrared, where the bSi alone remains transparent. Finally, a fabricated prototype device confirms the potential of III–V NWs combined with bSi for optoelectronic devices. Our results highlight the promise of III–V NWs on bSi for enhancing optoelectronic device performance on the low-cost Si substrates, and we believe that the new low-temperature NW growth regime advances the understanding and capabilities of NW growth.

## Introduction

In the recent years, III–V nanowires (NWs) have been shown suitable for the fabrication of various optoelectronic devices, including solar cells^1–4^, light emitting diodes (LEDs)^5–7^, lasers^8^ and photodetectors^9,10^. In order to reach high efficiencies, however, costly III–V substrates are presently required^1,2^, which can be prohibitive for practical device fabrication. Pathways for low-cost growth platforms are being explored intensively^9,11–14^. One of the most notable growth substrates is silicon, which allows epitaxial growth of III–V NWs^15^ and has promise for high-performance device fabrication^16^.

Regarding silicon devices, performance benefits can be acquired by using textured surface with pyramids such as black silicon (bSi)^17^, which is known for significant absorption enhancement in the visible wavelength range^17–19^. However, the infrared (IR) reflectivity is high even with black silicon, an issue that has been previously addressed by *e.g*. pyrolytic carbon coating^18^, Ag nanoparticles^20^ and oxide films^21,22^. Although these approaches provide high absorption in the IR wavelength region, their use in optoelectronics is limited since they are applicable only as passive components in devices. On the other hand, III–V NWs offer another means for efficient light trapping^1,2^ and consequently for broadening the absorption spectrum for bSi. Among the III–V materials, the band gap values far into the infrared region are available, allowing stronger absorption of the low-energy photons for which bSi remains transparent. Importantly, the semiconductor NWs can additionally act as active parts in device structures, and thus offer fabrication possibilities not available with the passive antireflection approaches.

The NW growth includes several subtypes with different advantages. For example, Au-catalyzed NWs are most thoroughly researched, and are suitable for a wide variety of materials. However, Au is thought to act as a possible deep-level contaminant that can hamper device performance. Alternatively, NWs can be seeded by *in-situ* deposited group-III particles. This self-catalyzed growth is advantageous (i) in the seed particle constituting only same elements as the NW crystal, (ii) in avoidance of seed particle deposition step prior to growth run and (iii) in the possibility of removing the seed particle prior to shell growth (*i.e*. by incorporating the group-III seed particle into the NW crystal). From device fabrication aspect, low-temperature growth is beneficial since NWs can then be incorporated on substrates that already contain *e.g*. doped regions that might undergo diffusion at high temperatures, or materials that could be damaged by heat. Additionally, crystal structure is important in attaining high-performance devices, where defect planes perpendicular to the NW axis can scatter charge carriers^23^, while NWs are often plagued by them. Despite years of intensive research, unexplored growth regimes remain untapped, and may hold possibilities for interesting, alternative NW growth modes.

Here, we explore for the first time the combination of black silicon and III–V nanowires, with expected benefits from the possibility for high quality crystal growth and photonic effects in nanowires. Black silicon is shown to be a suitable growth platform for various NW growth modes (Au-seeded and self-catalyzed) and III–V materials GaAs, InP and InAs. These materials are among the most suitable for photovoltaics, fast transistors, and optical communications. In particular, InAs with low band gap energy is well-suited for IR absorption, emission and detection, and additionally for absorption broadening on bSi where wavelengths above about 1.1 µm are not absorbed. For the self-catalyzed InAs NWs, we report a new isolated low-temperature growth regime, using the lowest reported MOVPE growth temperatures down to 280 °C for III–V NWs. In this interesting growth regime, consistent growth towards [-211] growth direction is demonstrated, where perpendicular defect planes are avoided altogether. Furthermore, negligible tapering is obtained for these NWs over >5 µm lengths, which is only rarely obtained for self-catalyzed NWs^24^. Finally, the suitability of bSi for III–V NWs is highlighted by demonstrating optoelectronic device fabrication. We hope that the growth of III–V NWs on bSi paves way for enhancing optoelectronic device performance on the low-cost Si substrates, and that the reported low-temperature NW growth regime broadens the capabilities and understanding of NW growth.

## Results and Discussion

### III–V nanowire growth on black silicon

**Figure 1:**
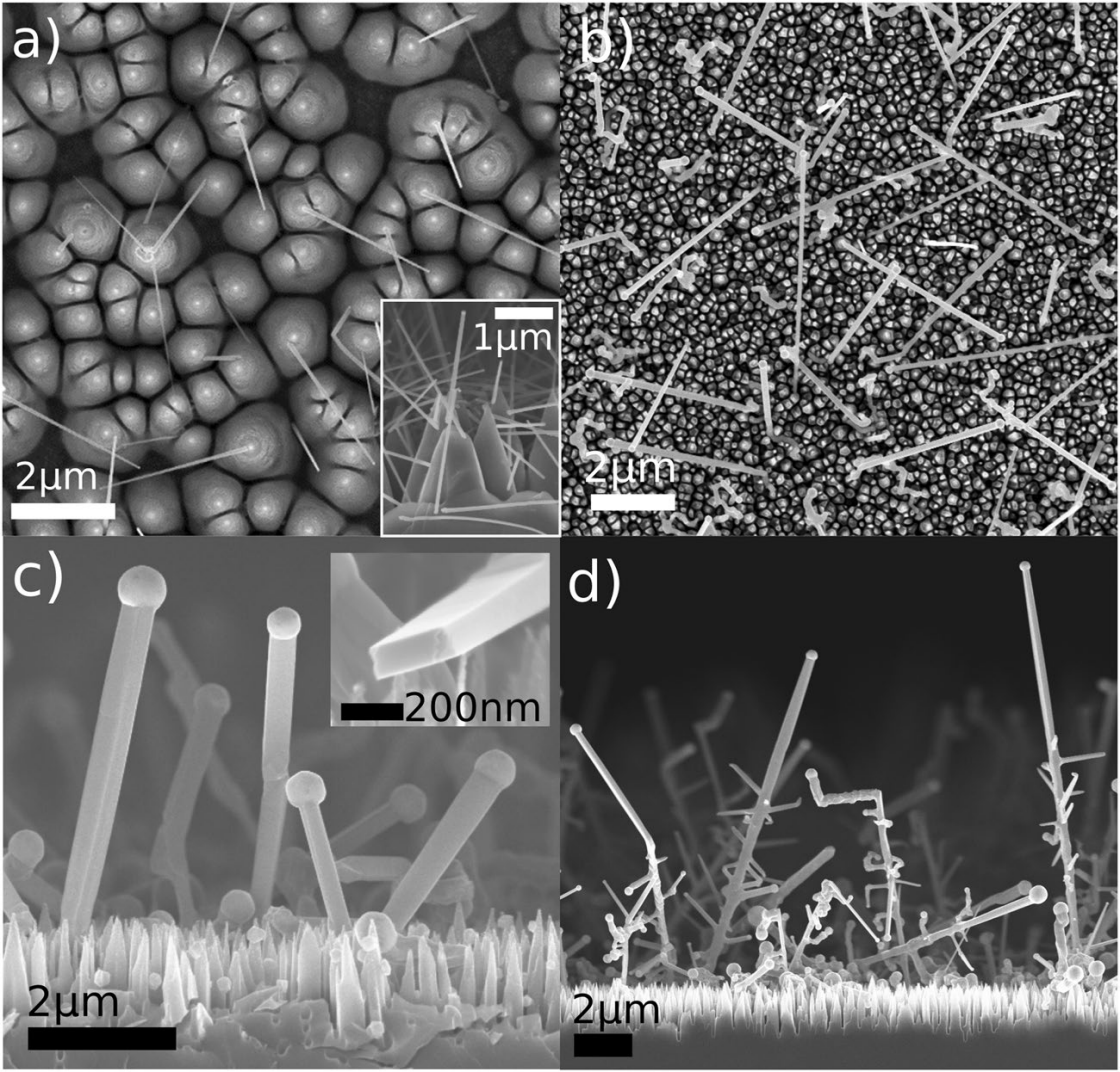
SEM images of various III–V NWs grown on (111) bSi: Au-seeded GaAs (**a**), In-seeded InP (**b**) In-seeded InAs at 365 °C with V/III = 2 (**c**) and heterostructured NW tree, *i.e*. InP NW trunks with InAs branches (**d**). The Au seed particles were deposited on HF-etched bSi in the (a) large picture and with intact native oxide in the (**a**) inset. Inset in (**c**) shows a cross-section view of a broken InAs NW.

Figure 1 shows SEM images of various III–V NWs grown on (111) bSi: Au-seeded GaAs NWs (Fig. 1a), and In-seeded InP NWs (Fig. 1b) and In-seeded InAs NWs (Fig. 1c). The Au seed particles were deposited from a colloid solution *ex-situ* and the In particles *in-situ* prior to NW growth. Clearly, the bSi substrate is suitable for the growth of various III–V NWs seeded by the most common particle types. With the *in-situ* seeded NWs, additionally heterostructure NWs comprising two different materials were fabricated. Figure 1d shows an example of InAs NWs grown on the sidewalls of InP NWs, and supporting information Figure S1 shows additional heterostructures, *i.e*. axial InP/InAs NWs and InP NWs branching from InAs NWs. Either with *in-situ* or *ex-situ* growth, the bSi surface retained the possibility of epitaxial NW growth after the ICP-RIE fabrication as evidenced by preferred growth directions in Fig. 1a,b. The epitaxial growth is beneficial for device fabrication due to better substrate/NW interface and more facile processing into devices. Notably, the directionality was observed only when the substrate was HF-etched, similarly to NWs grown on planar Si substrates^15^. Unlike on planar Si, the growth occurred at an angle from the substrate normal due to the slanted sidewalls of the bSi pyramids. Typically the NW growth occurs on (111) crystal planes, of which there are four on the Si[111] substrate, one towards the surface normal and three at a 45° angle, separated by 120° when viewed from above. Clearly, the preferential growth directions on bSi include several others besides the {111} directions, which is assumed to result from the wide selection of crystal planes exposed on the quasi-isotropically etched bSi pyramid sidewalls.

Interestingly, the NW growth can be tuned to occur predominantly on the tips of the bSi pyramids. For Au-seeded NWs, the bSi surface can be etched with HF to remove the native oxide and to render Si hydrophobic. As a result, solution containing Au nanoparticles rests on the pyramid tips, thus preventing the nanoparticles from reaching the valleys between the pyramids. Subsequently, during the NW growth run, the nanoparticles remain stationary and initiate the NW growth on their landing site. On the other hand, if the HF treatment prior to the Au nanoparticle deposition is omitted, the silicon surface remains non-hydrophobic, and the Au nanoparticle solution wets the bSi pyramids more effectively. This results in NW growth in all regions of the bSi surface, including the valley regions (Fig. 1a inset).

In case of the *in-situ* deposited In nanoparticles, the predominant growth on the tips is thought to result from faster growth nutrient supply to the bSi tips, whereas the gas exchange to the valleys between the pyramids occurs more slowly. This phenomenon is similar to shadowing effect often observed in core-shell NW growth, where the shells grow faster (i.e. thicker) near the NW tips^25^. Here, this explanation is supported by In droplets being larger near the tips of the bSi pyramids than between the pyramids. Figure S3 in Supporting Info shows the difference qualitatively. In addition, by measuring the diameter of 25–27 In droplets in the valley regions and the tip regions, the droplets near the tips were ~24% larger, giving further statistical evidence for the observation.

### Low-temperature growth of rectangular [-211] InAs NWs

**Figure 2:**
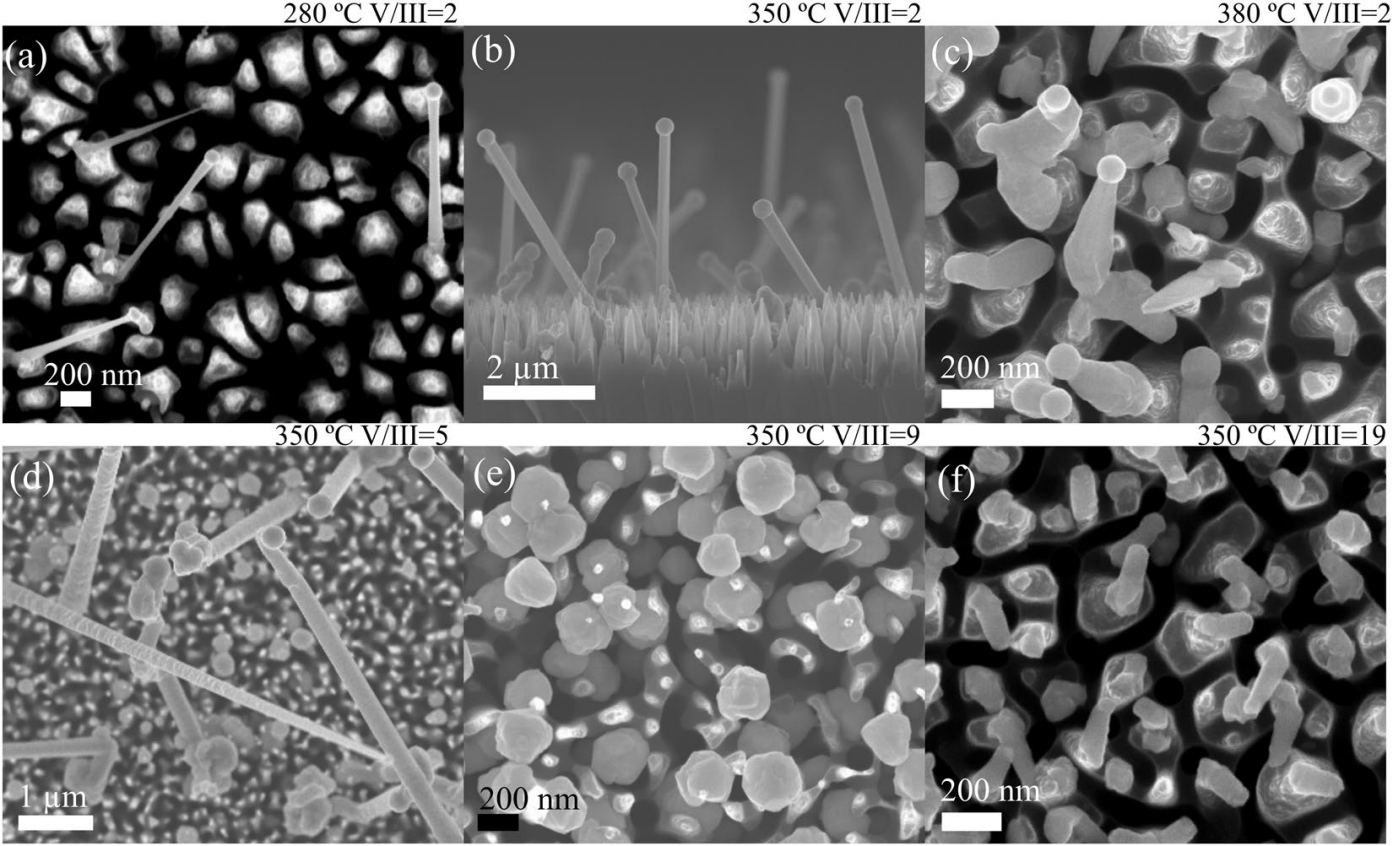
SEM images of self-catalyzed InAs NW growth parameter effects: growth with constant V/III = 2 results in straight NWs even at 280 °C (**a**), at 350 °C higher density and growth rate are acquired (**b**) and at 380 °C crawling growth and few hexagonal NWs (**c**). At constant growth temperature of 350 °C, V/III = 5 induces defects and tapering to some of the NWs (**d**) V/III = 9 results in growth of spheres, and (**e**) and V/III = 19 in short NW stubs (**f**).

Of the NWs reported here, the InAs NWs were grown using a record-low temperature of 280 °C for any MOVPE-grown NWs. Previously, InP NWs have been fabricated at 350 °C^26^, while InAs NWs are usually grown at 400 °C and above^27–33^. Below 400 °C, the InAs NW growth becomes typically impractically tapered^29,30^, although growth at 350 °C has also been reported^34^. The lower growth temperature means that the NWs can be applied to more numerous process flows, for instance, they are better suited for substrates which already contain doped regions (less diffusion), metallization (less impact on the metal-semiconductor interface) or other materials deposited on the growth substrate that be damaged by high temperature processing. The InAs NWs grown at different temperatures are presented in Fig. 2a–c.

The studied temperatures ranged from 270 °C to 380 °C. At 270 °C, no growth occurred due to insufficient temperature for precursor cracking. At 280 °C and above, straight NWs were obtained, although the precursor cracking remained limited up to ~310 °C resulting in low NW density. Interestingly, at the lowest growth temperatures the NWs grew on bSi but not on planar Si, which suggests enhanced precursor cracking on bSi resulting from larger surface area. Statistical analysis shows a rapid increase in the growth rate above 330 °C (Supporting Info Figure S2), which corresponds roughly to the temperature where the decomposition of both TMIn^35^ and TBAs^36^ increase rapidly. On the other hand, the droplet size (and thus the NW diameter) increases monotonously with the growth temperature (Figure S2). The optimal temperature range was deemed 340–365 °C, which resulted in straight NWs, fastest growth rate and highest NW density.

The growth at the low-temperature regime required additionally low V/III-ratio. At 350 °C, increasing the V/III ratio to 5 induced defects and shrinking diameter to some of the NWs (Fig. 2d). Already at V/III = 9, no NWs were obtained and the growth resulted in spheres instead (Fig. 2e). With V/III = 19, short stubs were observed, indicating that the conditions were again approaching those favorable for NW growth. Interestingly, when either temperature or V/III ratio was increased to towards typical III–V NW growth regime, the growth became worm-like or resulted in spheres. Therefore, the growth regime presented here is isolated both in terms of temperature and V/III-ratio from the typical growth of [111]-oriented III–V NWs. Similar parameter regions could exist for other III–V materials as well, opening new possibilities in tuning NW shape, dimensions and growth direction.

Further benefits for the presented InAs growth include reasonably high NW density that can be tuned by the pinhole density in the oxide, e.g. by immersing the Si growth substrate to H_2_O_2_ prior to droplet deposition (see Supporting Info Figure S3). This is crucial for NW devices yet often challenging with non-[111] NWs^27^. Further, the NW diameter has been predicted to be controllable by V/III ratio in self-catalyzed growth, and that constant NW diameter can be obtained under appropriate conditions^24^. Here, we observed that larger V/III ratio resulted in smaller average NW diameter, which corresponds to the theoretical prediction^24^. Additionally, the NWs had the same diameter regardless of the seed particle deposition time (*i.e*. size) when using the same V/III ratio (Supporting Info Figure S4). Therefore, the NW diameter is governed by the V/III ratio as predicted by theory^24^, and could be controlled depending on the final application. Additionally, a constant diameter was acquired for >5 µm long NWs, which is attributed to suppression of radial growth by the extremely low growth temperature.

**Figure 3:**
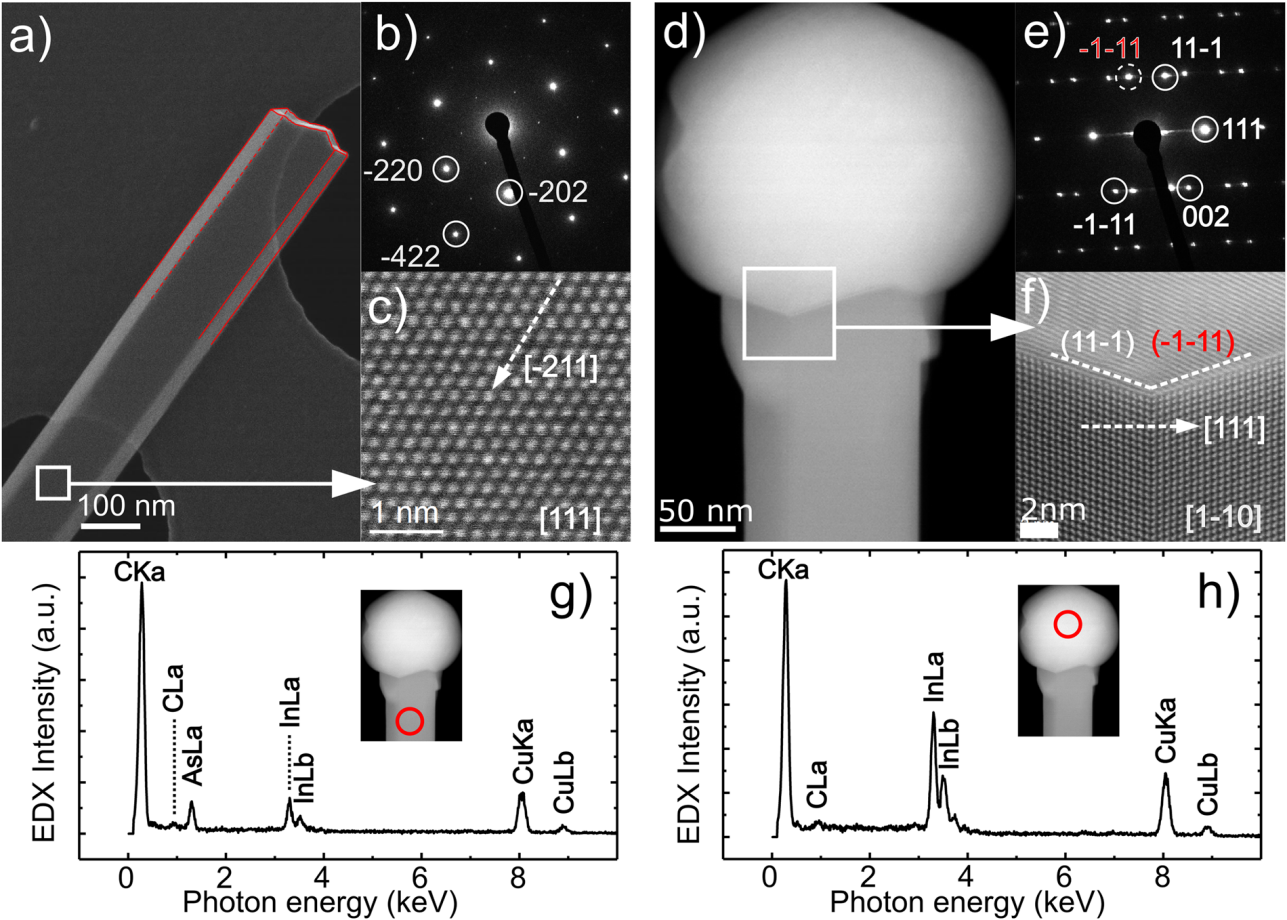
TEM and STEM analysis of rectangular InAs NWs with growth axis towards [-211] imaged both through long facet (**a**–**c**) and short facet (**d**–**f**). Corresponding electron diffraction patterns are shown in (**b**,**e**). EDX spectra are presented for the NW crystal (**g**) and In seed particle (**h**).

As suggested by SEM observations (Fig. 1c inset), the rectangular cross-section was evident also in the TEM and STEM analysis. First, STEM was used to identify the long and short sidewalls of the NWs. Figure 3a shows a tilted STEM view from a broken cross-section of an InAs NW, where the long and short facets can be clearly distinguished (emphasized with the red markings on the image). Tilting the NW long facet towards the electron beam, both the diffraction pattern and high-resolution images appeared hexagonal, corresponding to either zinc-blende (ZB) zone axis [111] or wurtzite zone axis [1000] (Fig. 3b,c). For a statistically significant analysis, roughly 10 NWs were imaged in TEM, with images and diffraction patterns taken towards both the long (Fig. 3c) and the short sidewalls (Fig. 3f). Further analysis on the diffraction patterns revealed ZB crystal structure, with the growth direction towards [-211] (instead of typical [111]^25,28,29,31,34^), and the sidewalls towards [111] (long facet) and [-1-10] (short facet). The growth direction of [-211] also results in the rectangular cross-section instead of the hexagonal type typically seen in [111]-oriented NWs. Supporting Info Figure S5 shows TEM and STEM images taken towards the [111] zone axis from a NW with a defect, showing additional periodicity resulting from the twin plane. Energy-dispersive X-ray spectroscopy (EDX) spectra shown in Fig. 3g,h show that the NW body was composed of InAs without additional detectable elements, and the seed particle consisted of only indium (the copper and carbon signals originate from the TEM grid). It should be emphasized that the NWs had consistent crystal structure and tunable areal density, whereas typical non-[111]-oriented growth results in various different growth directions^37,38^ or low NW densities^27^, rendering growth optimization or device fabrication challenging. Furthermore, the majority of the NWs in this study had few defects, which scatter charge carriers^23^ and are generally considered to affect negatively NW device performance. Therefore, the [-211]-oriented NWs reported here show a promise for device fabrication, especially since weaker impact on charge carriers is expected by the defects parallel to the NW compared to perpendicular defects.

Interestingly, the In seed particle shown in Fig. 3f forms an angle at the interface with the NW body. Therefore, the growth interface (i.e. the interface between the seed particle and the NW body) differs from the growth direction of [-211]. By analyzing the TEM images and the diffraction pattern (Fig. 3e,f), we can identify the growth interface as {111} planes that are typical for NW growth. Therefore, although the growth direction differs from the typical [111] direction, the growth occurs on {111} planes. Previously, increased In content in the seed particle (and thus increased amount of In available for the growth) has caused the NW growth to deviate from the typical [111] direction^27,39^. Also in this study, deviation from [111]-oriented growth is obtained with a low V/III ratio combined with low temperature. Therefore, the low-temperature and low-V/III regime represents an unexplored dimension in the tuning of NW growth. The cause for the deviation from [111]-oriented growth has been thought to result from the minimization of sidewall surface energies^27^. Similarly in here, the lowering of the surface energies is assumed to contribute to the deviation from the [111]-growth, which with the slanted (111) growth interface results in the most favorable combination.

### Reflectance

**Figure 4:**
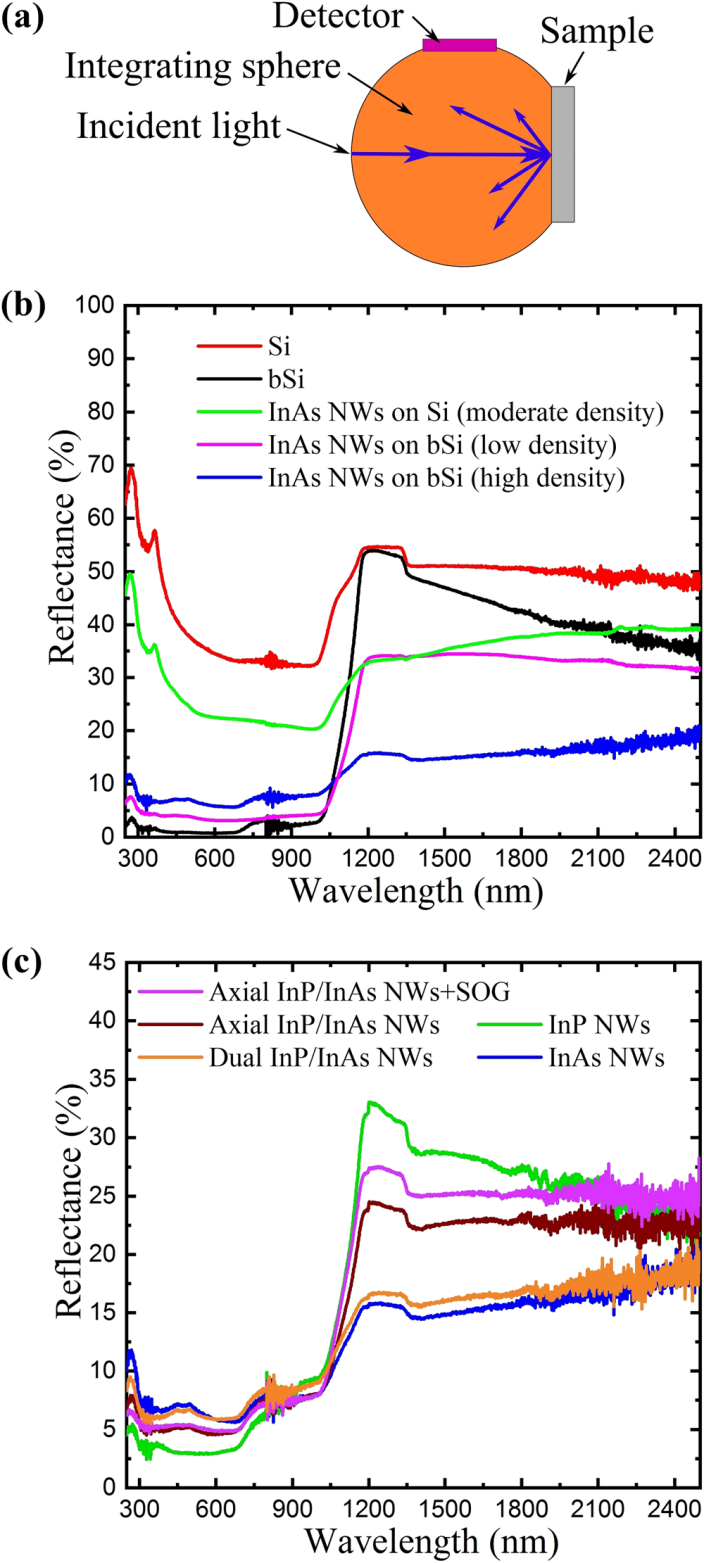
(**a**) Reflectance measurement schematic. (**b**) Reflectance spectra from planar Si and black Si substrates, both with and without InAs NWs. (**c**) Reflectance spectra from various NWs on bSi: InAs, InP, dual InP/InAs, and axial InP/InAs (before and after the application of spin-on-glass).

One of the aims of this study was to reduce the reflectance in the infrared regime, where bSi performs only moderately. In order to acquire the most complete picture of the reflectance, omnidirectional reflection was measured by an integrating sphere, as presented schematically in Fig. 4a. As shown in Fig. 4b, the bare Si and bSi surfaces behaved similarly to earlier reports^18,40^, *i.e*. bSi had very low reflectivity up to ~1000 nm, but only moderate reflectivity at longer wavelengths.

The relatively high reflectivity of bSi in IR region was remedied by the InAs NWs. The long-wavelength reflectance was reduced down to ~15% with relatively dense InAs NW forest, while retaining the reflectance below ~1050 nm at ~5–7%. Additionally, the reflectivity in different wavelength regions was found to depend on the density of the InAs NWs. Although relatively dense NW forest gave lowest reflectance on average over the studied wavelength range (SEM image shown in Fig. 1c), more sparse InAs NWs resulted in lower reflectivity below ~1050 nm (~3–4%) (SEM image shown in Figure S6a in Supporting Info). Therefore, depending on the application, the reflectivity at different wavelength regions can be tuned by NW density as demonstrated here, and additionally by different materials and NW dimensions. The suitability of bSi for low-reflectivity applications is further demonstrated by comparing InAs NWs on planar Si and bSi. As shown in Fig. 4b, the reflectance is significantly higher in visible wavelength range on planar Si even when using moderately high NW density (SEM image in Supporting Info Figure S6b). Comparing to earlier reports^41^, InAs NWs grown in ordered arrays have exhibited reflectivity values of ~3–30% at wavelength range 400–850 nm when measured with numerical aperture of 0.5 (i.e. with acceptance angle 60°). Here, the randomly oriented InAs NWs on bSi show similar or lower reflectance values even when measuring with integrating sphere that collects larger portion of the light, highlighting the capabilities of bSi as a growth substrate for low-reflectance NW applications.

Figure 4c shows the reflectance spectra from InAs NWs, InP NWs and dual InP/InAs NWs, all grown on bSi substrates. Interestingly, the InP NWs result in lower reflectance in the visible spectrum as compared to the InAs NWs, which further shows that the reflectance can be tuned via different NW types. One interesting notion is that the InP NWs reflection in the infrared range (below the InP band gap energy (1.344 eV or ~923 nm)) is lower (~25–30%) compared to bare bSi (~35–50%), indicating contribution from other photonic processes besides direct absorption. Especially scattering is expected from the sidewalls of the relatively long and broad NWs, and additionally smaller contribution is assumed from coupling between the IR photons and dense forest of antenna-like NWs. The role of the band gap effects and absorption is further highlighted by the IR reflectance reduction when InAs branches are added to the InP NWs. With these heterostructure NWs the reflectance is nearly as low as with InAs NWs.

In order to demonstrate an optoelectronic device, we have used spin-on-glass (SOG) for planarization and electrical isolation of the NWs from the bSi substrate. With the addition of electrical contacts, the InAs NWs (typically n-type without intentional doping^32,33^) on p-type bSi substrate can be used as a photodetector or a solar cell, which is used here for device demonstration. First, the processing effects on reflectance was studied by measuring reflectance before and after SOG application. As shown in Fig. 4c, addition of a SOG layer increases the reflectance by only ~1% in IR region and negligibly in the visible wavelength region. This indicates that with the combination of NWs and bSi, optoelectronic devices with high broadband absorption can be fabricated.

### Electrical characterization of InAs NWs on bSi

**Figure 5:**
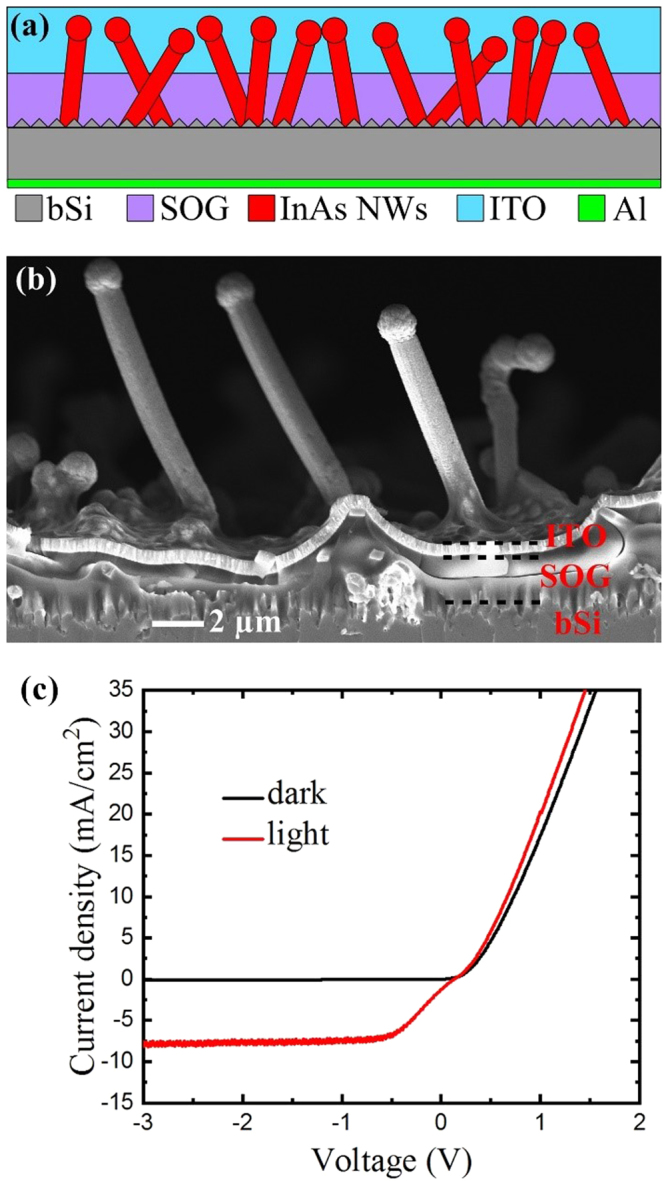
Schematic (**a**) and cross-sectional SEM micrograph of the NW device after fabrication (**b**). Current–voltage characteristics from InAs NW on bSi solar cells/photodetectors acquired in the dark and under illumination (**c**).

The device structure was finalized by sputtering transparent electrode, ITO, on the NWs and by evaporating aluminum to the bSi wafer backside. Several ITO contacts were fabricated on a single substrate by a deposition through a shadow mask with ~1 mm^2^ openings. The device structure schematic and a cross-sectional SEM image are shown in Fig. 5a,b. Figure 5c presents typical electrical characteristics of the n-type InAs NWs on p-type bSi at room temperature in the dark and under illumination. Little variance was observed in electrical behavior when measuring from different ITO contacts, which demonstrates consistency in the NW quality and in the processing scheme. In dark conditions, the device exhibits perfect diodic rectifying behavior with exponential increase in current at low voltage and ohmic behavior above the turn-on voltage. The junction I-V characteristic were non-linear and asymmetric with the forward threshold voltage of about 0.5 V and current on the order of hundreds of µA. While the reverse leakage current in the dark was small (below 3 µA at −3 V), the current was dramatically increasing under forward bias (reaching >1 mA at +2 V), indicating that good quality p-n junction was formed. The reverse breakdown occurred below −10 V (see Supporting Info Figure S7). Upon illumination with a halogen lamp, a clear response was observed in the electrical characteristics. The I-V curve shifted significantly downwards under illumination due to photogeneration and separation of electron–hole pairs. This shows that the structure can be used for photovoltaic or photodetection applications, and that the III–V NWs on bSi are suitable for optoelectronic device fabrication.

## Conclusions

In conclusion, we report for the first time on the combination of NWs and bSi for application in optoelectronic devices with high broadband absorption. In particular, we have investigated both *in-situ* and *ex-situ* seeded MOVPE growth of GaAs, InP and InAs NWs as well as axial and branched NW heterostructures on bSi. Evidently, bSi substrate is suitable for the growth of various III–V NWs seeded by the most common particle types and the NW growth occurs predominantly on the tips of the bSi pyramids. We found that low band gap energy InAs NWs on bSi reduced the IR reflectance significantly down to ~15%. Furthermore, the reflectance can be tuned via different NW types such as axial, dual or branched heterostructures. In addition, we demonstrated self-catalyzed growth of InAs NWs using a new low-temperature growth regime, where NWs were obtained at temperatures as low as 280 °C. The low-temperature growth can be extremely beneficial for a variety process flows where excessive heat is detrimental. This interesting growth regime also required low V/III ratio for NW growth. The NWs obtained in this regime had consistent growth towards [-211] growth direction where perpendicular defect planes are avoided altogether. Notably, low-temperature and low-V/III ratio InAs NWs exhibits tunable areal density, negligible tapering and consistent crystal structure with few defects. Finally, we have fabricated an optoelectronic device based on these InAs NWs and bSi. The I-V characteristics of the device confirmed the formation of p-n junction, good diodic behavior and a clear photoresponse. Overall, our results demonstrate that growth of III–V NWs on bSi has a high potential for use in photovoltaic or photodetection applications and that the reported low-temperature NW growth regime broadens the capabilities and understanding of NW growth.

## Methods

### Black silicon

The bSi was formed in a cryogenic, inductively coupled plasma reactive ion etcher (ICP-RIE; Plasmalab System 100, Oxford Instruments, UK) using the following optimized parameters: 40 sccm SF_6_, 18 sccm O_2_, 6 W forward power, 1000 W ICP power, −110 °C, 10 mTorr pressure and helium backside cooling.

### Nanowire growth

GaAs, InP and InAs NWs were fabricated on p-bSi (111) substrates inside a horizontal flow atmospheric pressure metal organic vapor phase epitaxy (MOVPE) system. Trimethylgallium (TMGa), trimethylindium (TMIn), tertiarybutylphosphine (TBP) and tertiarybutylarsene (TBAs) were used as precursors. The bSi surfaces were treated with hydrogen peroxide for 250 min in order to suppress the oxide pinhole density and thus to control the resulting NW density. Prior to the growth, the substrates were annealed *in situ* at 750 °C for 10 minutes under hydrogen flow. The annealing step was performed to modify the bSi surface, while it had no effect on the NW growth itself. For *ex-situ* seeded GaAs NWs, 40 nm diameter colloidal gold (Au) nanoparticles solution (BBI International, UK) was used as catalyst. The growth temperature was fixed at 470 °C for 150 s, which resulted in NW length of ~3 μm. During the growth, the TMGa and TBAs flows were 10.7 µmol/min and 270 µmol/min, respectively, and the nominal V/III ratio was 25. The *in-situ* seeded InAs and InP NWs were first grown from *in-situ* deposited In particles (using TMIn flow of 5.95 µmol/min for 15 s at 350 °C. The InAs NWs growth temperature was varied from 270–380 °C, and was fixed at 365 °C for the reflectance and device studies. The growth time of 2700 s resulted in NW length of ~7–9 μm. The TMIn flow was fixed at 5.95 µmol/min and the TBAs flow was varied from 9 to 113 µmol/min, with the nominal V/III ratio of 1.5 to 19. For the reflectance and device studies TBAs flow of 12.6 µmol/min was used. The InP NWs growth temperature was fixed at 380 °C for 210 s, which resulted in NW length of ~7–9 μm. The TMIn and TBP flows were 5.95 µmol/min and 678 µmol/min, respectively, and the nominal V/III ratio was ~114. Hydrogen was used as a carrier gas and the total reactor gas flow rate was ~ 5 l/min (slm). The growth temperatures reported in this work are thermocouple readings of the lamp-heated graphite susceptor, which are slightly higher than the real substrate surface temperature.

### Nanowire structure and morphology characterizations

Structural properties of the NWs were studied using scanning electron microscopy (SEM) (Zeiss Supra 40). High-resolution transmission electron microscopy (TEM) measurements were carried out with a JEOL 2200FS double aberration corrected FEG microscope operated at 200 kV. Elemental composition of the NWs was determined using a TEM integrated energy-dispersive X-ray (EDX) spectroscopy tool.

### Nanowire device fabrication

To prevent short-circuiting of the n contact to the p substrate, a spin-on-glass (SOG) buffer layer was applied. The SOG used in this work was ACCUGLASS T-512B provided by Honeywell. The SOG spun onto the substrate at 2000 RPM for 20 s and cured in furnace at 425 °C for 1 h in nitrogen atmosphere as instructed by the manufacturer. After repeating the procedure twice, the SOG formed a continuous 1 µm to 2 µm isolating layer over the substrate and covered only the base of the NWs. The bottom contact to the bSi substrate was obtained by evaporating 400 nm of Al and consequent annealing at 400 °C for 30 min. In order to form ohmic contact to InAs NWs, sulfur passivation of NWs in ammonium polysulfide was performed. The samples were soaked in deionized water (DIW):ammonium polysulfide solution (10:1) for 40 s at 45 °C, then rinsed in DIW and blown dried. The top contact to the NW tips was sputter coated with a 500-nm-thick indium tin oxide (ITO) layer and annealed at 300 °C for 3 min.

### Nanowire device characterization

The optical antireflection properties of the NWs grown on bSi were studied using Agilent Cary 5000 UV-Vis-NIR spectrophotometer equipped with 150 mm integrating sphere for diffuse reflectance measurements over the wavelength range of 250–2500 nm (0.5–4.9 eV). Halogen lamp was used as a light source and the reflectance was measured relative to the polytetrafluoroethylene (PTFE) reference plate, which has reflectivity above 96% between 200–2500 nm and more than 99% between 350–1800 nm. The photomultiplier tube (PMT) detector was used in the visible spectrum range and the Peltier cooled PbS detector was used in the IR range. The current–voltage (I-V) characteristics of the NW based devices were obtained using Karl Süss Probe station equipped with HP 4155 A semiconductor parameter analyzer.

## Electronic supplementary material

Supplementary information
