# Supplementary material for: III–V nanowires on black silicon and low-temperature growth of self-catalyzed rectangular InAs NWs
Source: Sci Rep. 2018 Apr 23;8:6410. doi: 10.1038/s41598-018-24665-9 (PMC5913270; doi:10.1038/s41598-018-24665-9)
Supplement: Supplementary file 1 — Supplementary information [file 41598_2018_24665_MOESM1_ESM.pdf]

# Supporting information for

# III-V nanowires on black silicon and low-temperature growth of self-catalyzed rectangular InAs NWs

*Tuomas Haggren[1], Vladislav Khayrudinov[1], Veer Dhaka[1], Hua Jiang[2], Ali Shah[1], Maria Kim[1] and Harri Lipsanen[1]*

[1]Department of Electronics and Nanoengineering, Micronova, Aalto University, P.O. Box 13500, FI-00076, Finland

[2]Department of Applied Physics, Aalto University, P.O. Box 15100, FI-00076, Finland

This study included the growth of two types of nanowire heterostructures: axial and branched NWs. Figure S1a shows NWs with lower segment from InP and top segment from InAs. These NWs were fabricated by switching the group-V precursor from tertirarybutylphosphine (TBP) to tertirarybutylarsine (TBAs) during the growth. The branched NWs shown in Figure S1b comprise InAs trunks on which thin InP NWs branches are grown. The InAs NWs were first grown from *in-situ* deposited In particles (using TMIn flow of 85 sccm for 15 s at 350 °C), and their growth was finalized by exhausting the In particle by switching off the group-III flow and keeping group-V flow on. Next, new In droplets were deposited by switching the group-V flow off and the In flow on for 30s. The 2$^{nd}$ seed deposition step was followed by InP NW growth by introducing TBAs to the reactor.

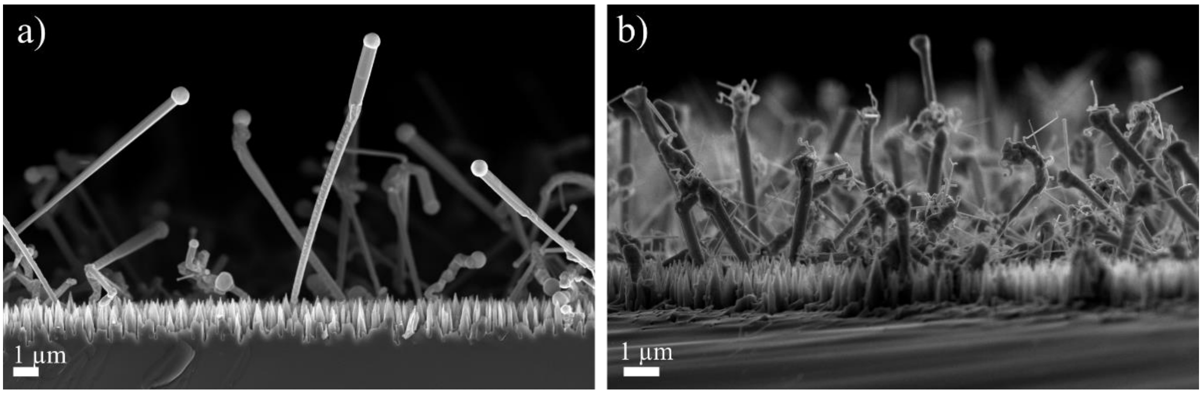


**Figure S1:** Two types of NW heterostructures: axial InP/InAs NWs (a) and InP NW 'trunks' with InAs NW 'branches'.

Statistical analysis was performed on the growth temperature effects on NW length and droplet size (i.e. NW diameter) (Figure S2). Ten NWs were studied at eight different growth temperatures. The higher temperatures resulted in longer NWs due to more efficient precursor cracking, with notable increase above ~330 °C. This corresponds roughly to the temperature at which the TMIn and TBAs decomposition rates increase rapidly. The droplet size and therefore NW diameter increased monotonously over the studied temperature range.

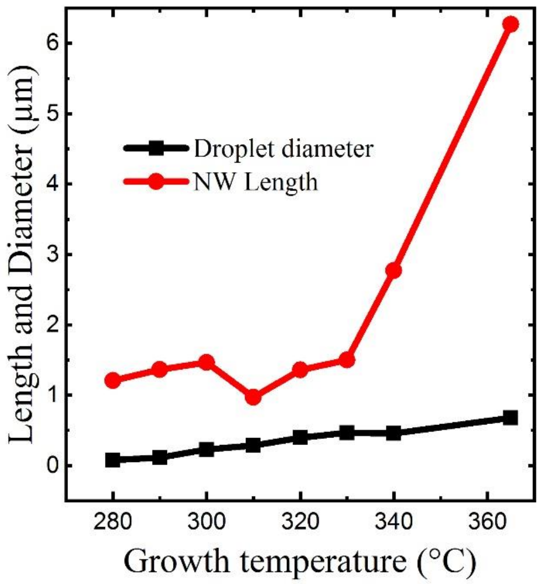


**Figure S2:** InAs NWs length and droplet diameter as a function of growth temperature.

The black silicon (bSi) surfaces were treated with hydrogen peroxide ($H_2O_2$) for 250 min in order to suppress the oxide pinhole density and thus to control the resulting NW density. Figure S3 shows indium droplets deposited on bSi for 15 s at 350 °C with TMIn flow of 85 sccm. Clearly, the non-treated surface (Fig. S3a) resulted in a higher density of smaller droplets compared to the treated surface shown in Fig. S3b. Especially with the lower droplet density in Fig. S3b, the droplet size was larger closer to the bSi pyramid tips compared to those in the valleys between the pyramids.

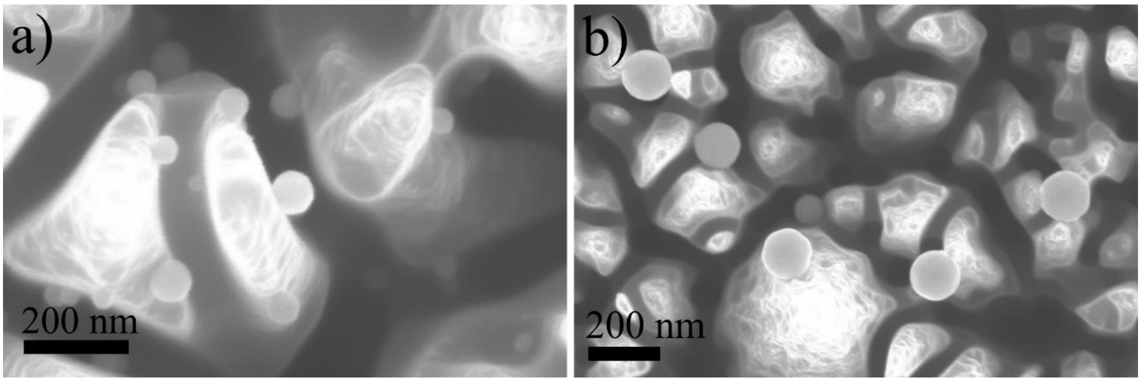


**Figure S3:** Indium droplets deposited on black silicon without (a) and with (b) $H_2O_2$ treatment for 250 min at room temperature.

The effect of In droplet size on the NW diameter is presented in Figure S4. The droplet deposition time was 15 s in Fig. S4a, and 45 s in Fig. S4b, *i.e.* the initial droplet volume was three times larger with 45 s deposition. However, since the NWs have similar diameters with both deposition times, it

can be concluded that the NW size is not significantly affected by the initial droplet size. Instead, it is assumed that the V/III ratio controls the NW diameter.

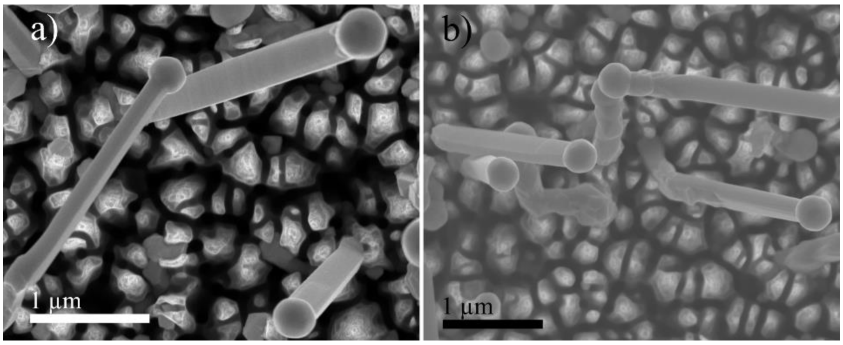


**Figure S4:** InAs NWs grown at 365 °C with V/III = 2 after In droplet deposition times of 15 s (a) and 45s (b).

The transmission electron microscopy (TEM) and scanning transmission electron microscopy (STEM) characterization revealed interesting periodicity in the defected NWs. In detail, these NWs were imaged towards a sidewall of the [-211] oriented NWs which corresponded to [111] zone axis. Figure S5 shows TEM and STEM images of NWs with twin planes perpendicular to the electron beam. Interestingly, the TEM image shows periodical brighter spots in addition to the InAs crystal. The periodicity is separated by a larger distance than that of the InAs crystal, and corresponds to the additional spots seen in the diffraction pattern. This additional periodicity are assumed to result from the twin planes that are perpendicular to the TEM / STEM imaging axis.

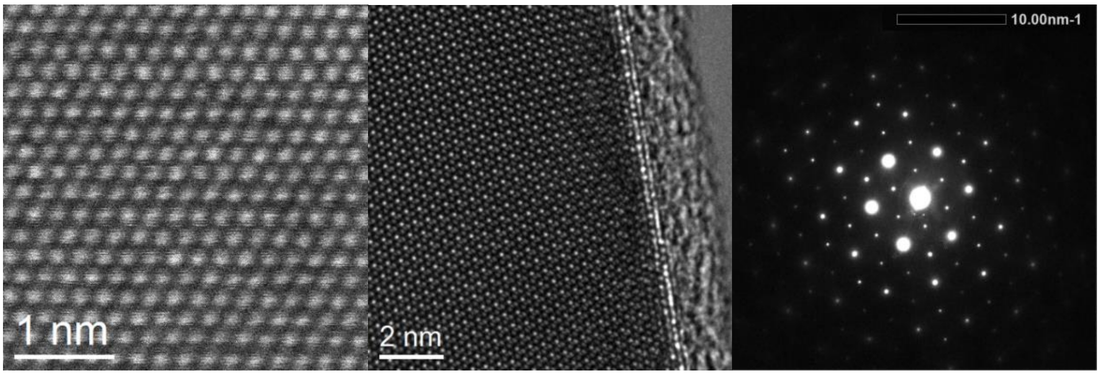


**Figure S5:** STEM (left) and TEM (middle) images of twin plane NW with additional periodicity, which is observed also in diffraction pattern (right).

Reflectance was measured from NWs on bSi and planar Si with different NW densities. Figure S6 shows SEM images from sparse InAs NWs on bSi and moderately dense InAs NWs on planar Si used in the reflectance measurements.

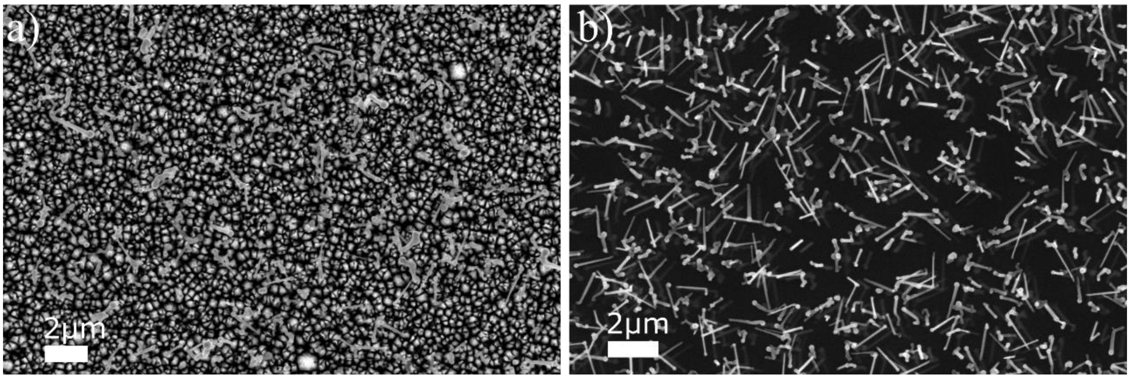


**Figure S6:** InAs NWs grown on bSi (left) and on planar Si (right).

A diode structure was fabricated using n-type InAs NWs and p-type black silicon. Figure S7 presents current-voltage behavior over a wide voltage range, where breakdown voltage occurs at around -10 V.

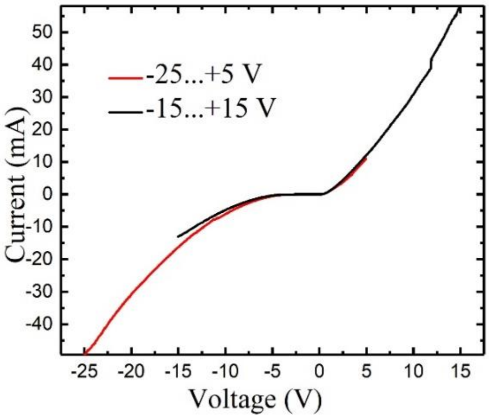


**Figure S7:** IV characteristics with higher bias from InAs NWs
